# Inequity aversion in dogs: a review

**DOI:** 10.3758/s13420-018-0338-x

**Published:** 2018-08-13

**Authors:** Jim McGetrick, Friederike Range

**Affiliations:** 1 Comparative Cognition Unit, Messerli Research Institute, University of Veterinary Medicine, Vienna, Medical University of Vienna & University of Vienna, Veterinärplatz 1, 1210 Vienna, Austria; 2 Konrad Lorenz Institute of Ethology, University of Veterinary Medicine, Vienna, Savoyenstraße 1a, 1160 Vienna, Austria

**Keywords:** Inequity Aversion, Dog, Cooperation

## Abstract

The study of inequity aversion in animals debuted with a report of the behaviour in capuchin monkeys (*Cebus apella*). This report generated many debates following a number of criticisms. Ultimately, however, the finding stimulated widespread interest, and multiple studies have since attempted to demonstrate inequity aversion in various other non-human animal species, with many positive results in addition to many studies in which no response to inequity was found. Domestic dogs represent an interesting case as, unlike many primates, they do not respond negatively to inequity in reward quality but do, however, respond negatively to being unrewarded in the presence of a rewarded partner. Numerous studies have been published on inequity aversion in dogs in recent years. Combining three tasks and seven peer-reviewed publications, over 140 individual dogs have been tested in inequity experiments. Consequently, dogs are one of the best studied species in this field and could offer insights into inequity aversion in other non-human animal species. In this review, we summarise and critically evaluate the current evidence for inequity aversion in dogs. Additionally, we provide a comprehensive discussion of two understudied aspects of inequity aversion, the underlying mechanisms and the ultimate function, drawing on the latest findings on these topics in dogs while also placing these developments in the context of what is known, or thought to be the case, in other non-human animal species. Finally, we highlight gaps in our understanding of inequity aversion in dogs and thereby identify potential avenues for future research in this area.

## Introduction

Inequity aversion refers to the resistance to inequitable outcomes and was initially proposed as an overarching explanation for seemingly contradictory behaviour of humans in economic experiments (Fehr & Schmidt, 1999). Inequity aversion implies that individuals are willing to forego material payoffs in order to achieve equity, and it is thought to ultimately maintain cooperation and protect individuals from exploitation. Two forms of inequity aversion are recognised: disadvantageous and advantageous inequity aversion. Disadvantageous inequity aversion occurs when an individual rejects inequity that is disadvantageous to itself, whereas the apparently less prevalent form, advantageous inequity aversion, occurs when an individual rejects inequity that is advantageous to itself and disadvantageous to its partner (Fehr & Schmidt, 1999). Throughout this review we use the term inequity aversion to refer exclusively to *disadvantageous* inequity aversion unless otherwise stated, as *advantageous* inequity aversion is typically not observed in non-human animal species (perhaps due to different neurological processing of these forms of inequity aversion; see Fliessbach et al., 2012; though, see Brosnan, Talbot, Ahlgren, Lambeth, & Schapiro, 2010 for an example of advantageous inequity aversion in chimpanzees).

Inequity aversion was restricted to the field of economics and cooperation in humans until the finding that capuchin monkeys (*Cebus apella*), like humans, reject inequitable outcomes (Brosnan & de Waal, 2003). In this study, a subject and a partner monkey in adjacent enclosures were both required to hand a token back to a human experimenter in exchange for a food reward. In the equity condition, both the subject and the partner received a piece of cucumber for successfully returning the token to the experimenter. In contrast, in the inequity condition, the partner received a higher value reward of grape while the subject continued to receive the lower value reward of cucumber for carrying out the same task. When faced with this inequity, subjects refused to return the token, or accept their food reward, on significantly more trials than in the equity condition.

Since the initial publication of inequity aversion in a non-human primate, the field has blossomed with studies claiming or dismissing the presence of an aversion to inequity in members of a steadily growing cohort of non-human animal species including chimpanzees (*Pan troglodytes*; Bräuer, Call, & Tomasello, 2006, Bräuer, Call, & Tomasello, 2009; Brosnan, Schiff, & de Waal, 2005; Brosnan et al., 2010; Hopper, Lambeth, Schapiro, & Brosnan, 2014; Ulber, Hamann, & Tomasello, 2017), bonobos (*Pan paniscus*; Bräuer et al., 2006, 2009), orangutans (*Pongo* spp.; Bräuer et al., 2006, 2009; Brosnan, Flemming, Talbot, Mayo, & Stoinski, 2011), gorillas (*Gorilla* spp.; Bräuer et al., 2006), long-tailed macaques (*Macaca fascicularis*; Massen, Van Den Berg, Spruijt, & Sterck, 2012), rhesus macaques (*Macaca mulatta*; Hopper et al., 2013), squirrel monkeys (*Samiri* spp.; Freeman et al., 2013; Talbot, Freeman, Williams, & Brosnan, 2011), cotton-top tamarins (*Saguinus Oedipus*; Cronin & Snowdon, 2008; McAuliffe, Shelton, & Stone, 2014; Neiworth, Johnson, Whillock, Greenberg, & Brown, 2009), owl monkeys (*Aotus spp.*) and marmosets (*Callithrix jacchus*; Freeman et al., 2013; *Callithrix spp.*; Mustoe, Harnisch, Hochfelder, Cavanaugh, & French, 2016), carrion crows (*Corvus corone corone*) and ravens (*Corvus corax*; Wascher & Bugnyar, 2013), New Caledonian crows (*Corvus moneduloies*; Jelbert, Singh, Gray, & Taylor, 2015), kea (*Nestor notabilis*; Heaney, Gray, & Taylor, 2017), rats (*Rattus norvegicus*; Oberliessen et al., 2016), and cleaner fish (*Labroides dimidiatus*; Raihani, McAuliffe, Brosnan, & Bshary, 2012). Many of these species that routinely engage in cooperative behaviours such as cooperative hunting or group defense, with unrelated individuals, seem to react negatively to unequal payoffs. In contrast, species that are social but that do not routinely cooperate with non-kin in such contexts, are less likely to react negatively to unequal payoffs, thereby providing support for the hypothesis that inequity aversion and cooperation coevolved (Brosnan, 2011; but see Bräuer et al., 2009; Freeman et al., 2013; Jelbert et al., 2015; McAuliffe et al., 2015; Raihani et al., 2012; Ulber et al., 2017).

Despite all the studies carried out to date, there are still numerous aspects of inequity aversion in animals that remain poorly understood and that warrant further investigation. First, limited attention has been afforded to the cognitive and emotional mechanisms underlying inequity aversion as well as its prerequisites. Second, although factors that influence inequity aversion have been identified in some species, we still lack an in-depth understanding of the development and expression of inequity aversion; such an in-depth understanding of these factors could help us to explain contradictory findings across research groups. Third, clear evidence demonstrating a relationship between cooperation and inequity aversion, that goes beyond correlational support at a phylogenetic level, is still required; therefore, broad conclusions regarding the function of the behaviour cannot yet be drawn. Fourth, whether inequity aversion is actually comparable, both in terms of its mechanisms and ultimate function, across the range of species studied to date is not yet known.

Moreover, in the larger framework of the proposed role of inequity aversion for the stabilisation of cooperative interactions, inequity aversion might play a dominant role in influencing whether animals engage in long-term reciprocal cooperative interactions and with which partners. The proximate mechanisms underlying reciprocity and partner choice in animals is currently one of the main topics in the study of the evolution of cooperation. Of particular intrigue is the question of whether individuals cooperate with specific partners in a calculated manner, involving complex cognition, or whether they choose cooperative partners based on simple emotional attitudes and development of long-term bonds. Developing an understanding of the mechanisms underlying inequity aversion in animals represents one approach to forming a more complete understanding of the mechanisms underlying reciprocity.

Although some studies have investigated factors influencing inequity aversion (see, for example Brosnan et al., 2015; Talbot et al., 2018), in general, studies on inequity aversion in non-human animals tend to rather opportunistically test whether a particular species reacts aversively to inequity without further investigating the various aspects outlined above. In contrast, several studies have been carried out over the past decade with dogs, attempting to understand the underlying mechanisms and function of inequity aversion. Apart from deepening our knowledge and understanding of canine cooperation and domestication, these studies also represent potentially novel insights into various aspects of inequity aversion in other non-human animals. Furthermore, dogs are a particularly interesting species to study in the context of inequity aversion given that they are arguably the only non-human animal species for which the exchange task with humans is ecologically or socially valid.

Here, we critically evaluate the current evidence for inequity aversion in dogs in light of both the latest findings and the issues that have arisen in studies of inequity aversion in other species. Additionally, we discuss the possible cognitive underpinnings of inequity aversion, and its ultimate function, potentially providing insights into the behaviour in other non-human animal species. Finally, for each of the aspects discussed, we identify emerging questions and gaps in our knowledge, which ultimately highlight avenues for future research.

## Inequity aversion in dogs

Range, Horn, Virányi, and Huber (2009a) demonstrated a negative reaction to an inequitable distribution of food rewards, in pet dogs. In this study, a similar design to that used to demonstrate inequity aversion in capuchin monkeys was employed. Pairs of dogs from the same household were seated next to each other while a human experimenter knelt before them asking the dogs, alternately, to give their paw. Upon successfully performing this task, dogs were rewarded (or not, depending on the condition; see Table 1) from a bowl of food placed directly in front of the experimenter, equidistant from both dogs. Bread was used as a “low value” reward (LVR) and sausage was used as a “high value” reward (HVR).

**Table 1.** All conditions tested in the paw and buzzer tasks with dogs in the various studies

**Condition**	**Subject**	**Partner**
***SOCIAL CONDITIONS***		
**Equity (ET)**^**a,b,c,d**^	LVR	LVR
**Quality Inequity (QI)** ^**a,b,c,d**^	LVR	HVR
**Effort Control (EC)** ^**a**^	LVR	LVR
**Food Control (FC)** ^**b,c,d**^	HVR moved, LVR given	HVR moved, LVR given
**Reward Inequity (RI)** ^**a,b,c,d**^	No reward	LVR^e^
**Social Control (SC)** ^**a,f**^	No reward	No reward
***ASOCIAL CONDITIONS***		
**No-Reward (NR)** ^**a,b,c,d**^	No reward	Not present^g^
**Assessment Control (AC)** ^**a,b,c,d**^	LVR	Not present^g^

LVR, low value reward; HVR, high value reward

^a^Range et al., 2009a (paw task)

^b^Brucks, Essler, Marshall-Pescini, & Range, 2016 (paw task)

^c^Essler, Marshall-Pescini, & Range, 2017 (buzzer task)

^d^Brucks et al., 2017 (buzzer task)

^e^In Range et al. (2009a) a piece of LVR was given to the partner; however, in the other three studies, HVR was given

^f^A reward was lifted from the food bowl after each time a dog gave its paw; it was then placed back in the bowl

^g^To control for movement of the food that occurs when feeding the partner in the social conditions, in the asocial conditions a piece of LVR was moved to the partner’s empty position on each trial in Range et al. (2009a), Brucks et al. (2016), and Brucks, Marshall-Pescini, et al. (2017); however, a piece of HVR rather than LVR was moved in the NR condition in Essler et al. (2017)

In the equity condition (ET), both the subject and partner received the LVR each time they gave the paw (see Table 1). In contrast, in one of the inequity conditions (quality inequity [QI]), the partner received a piece of HVR for giving the paw, while the subject received the LVR for performing the same paw-giving action. This latter condition, therefore, created inequity in the quality of rewards received by the two individuals and was analogous to the inequity condition experienced by capuchin monkeys in Brosnan and de Waal’s (2003) study. Unlike capuchin monkeys, however, dogs did not display any negative reaction to this inequity; they continued giving their paw for as many trials in the QI condition as the ET condition. Moreover, similarly to chimpanzees (Brosnan et al., 2010), but differently from capuchin monkeys (Brosnan & de Waal, 2003; van Wolkenten, Brosnan, & de Waal, 2007), there was no evidence that subjects reacted to inequity due to differences in effort; they continued working even when the partner received a reward for free and they themselves had to work for a reward (effort control [EC]).

Subjects refused to continue giving their paw, however, in the reward inequity (RI) condition. Here, they received no reward for giving their paw while the partner received the LVR for the same task. The number of trials in which subjects gave the paw in this condition was significantly lower than that of the ET condition. Additionally, to rule out the possibility that subjects stopped giving their paw simply because they did not receive a reward, they were tested with a “no-reward” (NR) condition in which they received no reward for giving the paw but no partner was present (a piece of LVR was moved to the partner’s empty position before being placed back in the bowl on each trial, to control for movement of the food in the RI condition). The number of times the subjects gave the paw in the RI condition was significantly lower than that of the NR condition suggesting that the presence of a rewarded partner, rather than the mere lack of reward, was the reason for the refusal to continue. In addition to the refusal to continue working in the RI condition, subjects also exhibited a significantly greater number of stress signals (e.g. lip-licking, yawning, scratching, avoiding the gaze of the partner), and required a greater number of paw commands, in the RI than in the ET and NR conditions.

In a second experiment, to rule out the possibility that dogs respond differently to the lack of reward in the presence of a partner, Range et al. (2009a) exposed naive subjects to two conditions: the RI condition and a new social control (SC) (see Table 1). This SC condition represented equity as a partner was present but neither dog received a reward for giving the paw. A trend emerged whereby dogs refused to give their paw earlier in the RI condition than in the SC condition. Although this was not a significant difference, there were significantly more stress signals and paw commands in the RI condition than the SC condition. Combined, these results suggest that dogs’ responses are driven by a sensitivity to inequity, rather than social facilitation or simply the lack of reward, alone. However, given the lack of a response to inequity in the quality of rewards received in the QI condition, Range et al. (2009a) concluded that dogs possess a *primitive* form of disadvantageous inequity aversion.

### Studies supporting the claim of inequity aversion in dogs

Issues surrounding replicability and reproducibility of research findings in comparative psychology is of growing concern, sometimes even referred to as a “replication crisis” (see Ioannidis, 2005; Open Science Collaboration, 2015; Stevens, 2017). Replicability issues have consistently plagued the study of inequity aversion with other research groups failing to replicate Brosnan and de Waal’s (2003) and Brosnan et al.’s (2005) findings of inequity aversion in capuchin monkeys and chimpanzees respectively (Bräuer et al., 2006; Dubreuil, Gentile, & Visalberghi, 2006; Roma, Silberberg, Ruggiero, & Suomi, 2006). In many of these studies, however, a subject and partner were handed rewards for free; the individuals did not have to invest any effort in a task such as exchanging a token. It has been argued that such an investment of effort is crucial for eliciting responses to inequity (Brosnan & de Waal, 2006; Dindo & De Waal, 2007), and evidence from inequity experiments with chimpanzees (Brosnan et al., 2010) and long-tailed macaques (Massen et al., 2012) supports this. Nevertheless, even with the inclusion of a task, a number of studies from different groups have failed to demonstrate inequity aversion in capuchin monkeys and chimpanzees (e.g. Bräuer et al., 2009; McAuliffe et al., 2015; Silberberg, Crescimbene, Addessi, Anderson, & Visalberghi, 2009; Ulber et al., 2017). Thus, replication remains an issue in the study of inequity aversion in non-human animal species. Importantly, in the context of this review, the finding that dogs display a primitive form of inequity aversion has now been replicated in two additional studies.

#### Paw task

**Fig. 1:**
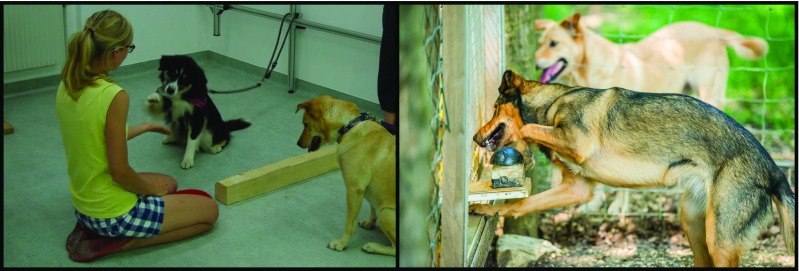
Paw task (left) and buzzer task (right)

In a replication of the original paw task study (see Figure 1), Brucks et al. (2016) tested 32 naïve subjects and obtained the same pattern as Range et al. (2009a). Subjects gave their paw significantly fewer times, exhibited more stress signals, and required a greater number of paw commands per trial, in the RI condition compared with the ET and NR conditions. In fact, subjects gave their paw even fewer times in the RI condition of this study than that of Range et al. (2009a). The difference in the strength of the negative reaction between the two studies is most likely due to a difference between the two studies in the quality of the reward used in the RI condition. Brucks et al. (2016) rewarded the partner with HVR in the RI condition, meaning that the subjects experienced a greater degree of inequity than in Range et al. (2009a) in which only LVR was used; the greater degree of inequity in Brucks et al. (2016) may have elicited a stronger reaction.

Brucks et al. (2016) also included an extra “food control” condition (see Table 1), the results of which further support the conclusion of inequity aversion. We will discuss the significance of this control later.

#### Buzzer task

Essler et al.’s (2017) recent comparison of inequity aversion in pack-living domestic dogs, and captive wolves (raised in a similar manner to the pack-living dogs), is the second study to confirm inequity aversion in dogs. In this study, both dogs and wolves were tested using a paradigm similar to that used in the paw task studies; however, the task itself differed. Rather than being asked to give their paw, the subject and partner in adjacent enclosures were each required to press a buzzer on command in return for food rewards (see Figure 1). Distribution of food rewards in this study matched that in Brucks et al. (2016) and Range et al. (2009a); a human experimenter took rewards from a bowl that contained both the LVR and HVR, and handed them to the appropriate individual. The results followed a similar pattern to those of the two paw task studies: dogs pressed the buzzer significantly fewer times in the RI condition than in the ET or NR conditions.

Additionally, Essler et al. (2017) demonstrated, for the first time, that wolves (*Canis lupus*), like dogs, respond negatively to inequity. Wolves in the buzzer task study also pressed the buzzer fewer times in the RI condition than in the ET and NR conditions. Thus, inequity aversion in dogs is unlikely to be a consequence of domestication. Interestingly, a significant difference was found between the QI and ET condition when the performances of the wolves and dogs were combined. Two out of nine dogs, and three out of nine wolves, stopped pressing the buzzer in the QI condition; yet, these individuals all continued until the maximum count of 30 buzzer presses in the ET condition. The wolves also had to be asked significantly more often to comply with the command to press the buzzer in the QI condition compared with the ET condition. At the very least, the findings of Essler et al. (2017) (buzzer task) and Brucks et al. (2016) (paw task), combined, strengthen the original claims of a primitive form of inequity aversion in dogs.

### Studies not supporting the claim of inequity aversion in dogs

#### Choice-of-trainer paradigm

Despite the support for Range et al.’s (2009a) original finding, in the above-mentioned studies, Horowitz (2012) failed to find evidence for inequity aversion in dogs, using a different paradigm. Here, subjects were exposed to two different pairs of trainers. In both pairs, one trainer rewarded the subject and partner equally by giving them the same amount of food (one piece each), while the other trainer distributed rewards unevenly. In one of the combinations of trainers, the “unfair” trainer provided the subject with one piece of food and the partner with no food, while in the second combination, the “unfair” trainer provided the subject with one piece of food and the partner with three pieces of food. Thus, the two unfair trainers created disadvantageous inequity and advantageous inequity respectively, from the point of view of the subject.

Following a familiarisation period, dogs had a test trial in which they could choose between the fair trainer and unfair trainer who had created either disadvantageous or advantageous inequity. When subjects were given the choice of a fair trainer or the unfair trainer who had created *advantageous* inequity from the subject’s own perspective, they had no preference for either trainer. Furthermore, when given the choice between the fair trainer and the trainer who had previously created *disadvantageous* inequity, they chose the latter. This suggests that the dogs have a preference for inequity that is disadvantageous to themselves. These results, therefore, seem to contradict the earlier findings by Range et al. (2009a) that dogs are averse to disadvantageous inequity.

This result is particularly unusual as it demonstrates that dogs choose the option that puts them at the greatest disadvantage compared with a partner. However, this result can be explained by a major confound in the experiment design: as Horowitz (2012) pointed out, the most plausible explanation for this finding is that subjects chose this trainer based on the fact that, during the experience phase, they had a greater number of food rewards than the fair trainer, thereby offering the potential to better reward the subject in the future. Nevertheless, even if this confound was not present, at least three others exist that reduce the study’s comparability with Range et al. (2009a). First, this was a choice task rather than a task in which inequity was forced upon the subject; thus, the social and emotional context is likely to differ significantly from those tasks that elicit negative responses to inequity. For example, although unlikely, one could argue that subjects in this study were making the prosocial choice, as previously shown in dogs (Quervel-Chaumette, Dale, Marshall-Pescini, & Range, 2015). Second, the subject was required to make their choice in the absence of their partner, which may, in fact, have reduced the social relevance of the task. Third, the trainers did not present food during the test trial, potentially making it more difficult for subjects to associate them with differing payoffs. In conclusion, we do not consider the results of this study to represent a substantial challenge to previous conclusions of a primitive form of inequity aversion in dogs.

#### Buzzer task with pet dogs

Perhaps more challenging to the claim that dogs are inequity averse, is the recent failure to demonstrate a negative response to inequity in pet dogs, using the same buzzer task used with pack-living dogs and wolves (Brucks, Marshall-Pescini, et al., 2017; Essler et al., 2017). Brucks, Marshall-Pescini, et al. (2017) carried out two versions of the buzzer task: in an “experimenter absent” version of the task, no human experimenter was visible and no verbal commands were given to the dogs; buzzers and food rewards were pushed into the dogs’ enclosures by two experimenters hidden behind a curtain. In contrast, in an “experimenter present” version of the task, a third experimenter sat in front of the curtain issuing commands to the dogs to press the buzzer. This third experimenter also delivered the rewards by pushing them, on containers, into the enclosures.

In this study, pet dogs stopped pressing the buzzer in the RI condition, and the number of times they pressed the buzzer was significantly lower than in the ET condition (Brucks, Marshall-Pescini, et al., 2017). However, there was no difference in the number of buzzer presses between the RI and NR conditions regardless of whether an experimenter was visible or not, indicating that refusals to continue were simply due to the absence of reward and were not related to comparison with what the partner received. Although dogs exhibited significantly more stress signals in the RI condition than the NR condition in both versions of the task, the overall result is not in line with previous conclusions that dogs are inequity averse. In general, the lower performance of dogs in the NR condition of this study compared with previous paw task studies (Brucks et al., 2016; Range et al. 2009a) suggests dogs’ motivation to perform this buzzer task without reward was lower. Motivation to participate in this task may, in fact, have been too low to allow a negative response to inequity to emerge.

A number of factors might have influenced the motivation of the dogs to perform this task compared with the previous paw and buzzer tasks. First, a single bowl full of food was always present and visible to subjects in the previous studies (Brucks et al., 2016; Essler et al., 2017; Range et al. 2009a). In contrast, in this buzzer task study, each dog was presented with two single pieces of food (one LVR and one HVR) on each trial, but a bowl full of food was never visible. A bowl full of food may be a stronger motivator for the dogs, than single pieces of food. Second, pet dogs in this study performed the task from within enclosures (Brucks, Marshall-Pescini, et al., 2017); although pack-living dogs were also restricted to separate enclosures for this task and were inequity averse (Essler et al., 2017), pet dogs may have been less familiar with being surrounded by fences and this might have affected their motivation to engage with the task when unrewarded. Furthermore, the physical barrier between the human experimenter and the participants, and the lack of physical contact, might have contributed to reduced motivation to continue with the task without rewards.

An alternative possibility, however, is that the motivation to perform the task was over-exaggerated in the NR condition of the previous inequity studies. In the NR condition of the previous three studies demonstrating inequity aversion in dogs, rewards were lifted up on each trial, in front of the subject, and moved to the partner’s empty position, before being moved back to the bowl (Brucks et al., 2016; Essler et al., 2017; Range et al. 2009a). This simple action was included to control for the movement of the food that occurs in the RI condition when feeding the partner. However, removing the single variable of a partner might have altered the situation in unintended ways. The aimless movement of food by the human might have been difficult for the subjects to interpret; they may, for example, have perceived these movements as offers or as indicative of the attainability of rewards. Thus, the NR control condition may have inadvertently enhanced the dogs’ expectation that they could be rewarded and, consequently, enhanced their willingness to work without a reward. In the NR condition of the buzzer task study with pet dogs, a reward was pushed into the partner’s empty enclosure on a container on each trial; however, this reward was behind the fence at all times, at a distance from the subject, and it was not moved in the human’s hand, which might have further reduced its salience. The same potential expectations created in the NR condition of the three other studies might not have been created here, thereby explaining the lack of inequity aversion obtained by Brucks, Marshall-Pescini, et al. (2017).

If this latter hypothesis is true, it would suggest that the primitive form of inequity aversion shown in dogs was a false positive and that refusals to work in the RI condition were simply responses to the lack of reward. Currently, this seems somewhat unlikely given the cases of quality inequity aversion already mentioned (Essler et al., 2017), the consequences of unequal rewarding on later social interactions (Brucks et al., 2016; see section on ultimate function, below), and the observation that even in the buzzer task of Brucks, Marshall-Pescini, et al. (2017) pet dogs displayed more stress signals in the RI condition than the NR condition despite no difference in performance between these conditions. Therefore, we continue on the assumption that dogs are, indeed, inequity averse. Nevertheless, this recent and unexpected discrepancy across studies demands further investigation.

### Summary

To summarise, domestic dogs have been shown to display a *primitive* form of inequity aversion in two different tasks, across three different studies. This does not seem to be a result of domestication as their closest living relatives, wolves, exhibit a similar response. To date, two studies have failed to replicate the finding of inequity aversion in dogs. However, one of these studies, a choice task, is confounded by aspects of the experimental design. The second, a buzzer task with pet dogs, represents a legitimate challenge to the claim of inequity aversion in dogs and, therefore, provides a need for further research; however, even the results of this study do indicate a likely aversion to inequity.

## Mechanisms

In this section we begin by addressing the numerous hypotheses that have been put forward as alternative explanations for negative responses to inequity in exchange paradigms with primates, and we indicate how these have been ruled out in the case of dogs. Assuming inequity aversion, rather than these alternative explanations, accounts for the negative responses of dogs to inequity, we then explore the possible cognitive prerequisites for, and mechanisms underlying, inequity aversion. Finally, we discuss the variety of factors identified to date that might influence inequity aversion, including the factors that might explain the lack of a response to quality inequity in dogs, while we also discuss the potential influence of humans on inequity aversion in pet dogs.

### Alternative explanations for negative responses to inequity in the exchange paradigms

#### The mere presence of the reward

Inequity aversion in non-human animals has received extensive criticism and generated heated debates ever since it was first reported in capuchin monkeys. Many alternatives to inequity aversion have been proposed that could account for the responses of capuchin monkeys to inequity. For example, Wynne (2004) argued that the capuchin monkeys may have been responding to the mere presence of the better reward of grape rather than the fact that their partner was receiving it and they themselves were not; grapes were not present in the equity condition in which the monkeys continued to work and accept cucumber (Brosnan & de Waal, 2003). In the paw and buzzer tasks with dogs, the possibility that the mere presence of rewards influenced responses was ruled out by ensuring that, in all conditions, both reward types were always visible to both dogs.

#### Food expectation

A related alternative hypothesis is that of “food expectation” (Bräuer et al., 2006; Dubreuil et al., 2006; Neiworth et al., 2009). According to the food expectation hypothesis, individuals expect they will receive the better quality reward, and their subsequent refusals to continue working, or to accept their rewards, result from a violation of expectation when they do not receive the better quality reward. Food expectation is, however, difficult to rule out as it is not possible to determine what the individual actually expects. Also, food expectation, or more specifically, socially influenced food expectation, might be an important factor contributing to subjects’ perception of inequity (e.g. Brosnan et al., 2011; Brosnan et al., 2010; Hopper et al., 2014). For example, a subject might expect that they will receive, or should receive, the better reward *because* their partner receives it. Thus, in order to rule out food expectation as an alternative explanation for inequity aversion, it is important to distinguish individual expectation, based on what is present in the environment, from socially facilitated expectation.

Various experiments with primates have attempted to control for individual expectations by either inducing incorrect expectations or making clear what the subject was about to receive by holding up specific rewards before token exchanges (Brosnan et al., 2010; Hopper et al., 2014; van Wolkenten et al., 2007). Although there was evidence for responses based on individual food expectation in some cases, these studies demonstrated that inequity aversion can occur independently of food expectation.

In the initial paw task study with dogs (Range et al. 2009a), food expectation should have been the same in the reward inequity condition in which dogs refused to continue working, and the no-reward control condition. Food expectation cannot, therefore, account for responses of dogs to inequity (though, see the discussion on the buzzer task, above, for a possible exception). Furthermore, in later inequity studies demonstrating inequity aversion in dogs (Brucks et al., 2016; Essler et al., 2017), a food control condition was incorporated into the experiment design (see Table 1). In this condition, the experimenter lifted a piece of the higher value reward up, after the subject gave the paw or pressed the buzzer, and subsequently placed this reward back in the bowl, giving the subject the lower value reward instead. This condition did not elicit a negative response in either of these two studies; however, pet dogs exhibited a negative reaction to a similar food control condition in the buzzer task, in which no inequity aversion was observed (experimenter absent version; Brucks, Marshall-Pescini, et al., 2017).

#### Successive negative contrast

Another prominent, alternative hypothesis to inequity aversion, postulates that capuchins refused to accept their food reward, or to continue with the task, in Brosnan and de Waal’s (2003) study, due to successive negative contrast (i.e. the “frustration effect”). Successive negative contrast refers to the reduction in instrumental or consummatory response, exhibited by many mammal species, following an unexpected downshift in reward quality or quantity (Cuenya et al., 2015; Flaherty, 1982, 1999; Papini, 2014; Tinklepaugh, 1928). Roma et al. (2006) highlighted the possibility that this reward scheduling effect could explain capuchin monkeys’ responses, arguing that, in Brosnan and de Waal’s initial study, monkeys that began the study as the partner, experienced receiving the better quality reward of grape *before* they experienced the inequity condition as a subject, in which their reward was downgraded to cucumber. Additionally, Roma et al. (2006) provided evidence, from their own study with capuchin monkeys, that prior receipt of grape can reduce the tendency of subjects to accept cucumber, compared with subjects that only ever receive cucumber. However, Brosnan and de Waal (2006) rejected this hypothesis. Reanalysing their original data, they reported that there was no difference, in the response to inequity, between subjects that had *not* previously received a grape, as a partner in the study, and those that *had* received a grape in a previous condition.

Range et al. (2009a) ruled out the “frustration effect” as an explanation for responses to inequity in the paw task with dogs, by providing subjects with a piece of the HVR before the RI and NR conditions to ensure that subjects experienced an equal downshift in rewards in both unrewarded conditions. Furthermore, neither the RI nor the NR condition was tested as the first condition, to ensure that all subjects had received rewards in the experimental context before receiving nothing for the same task (Brucks et al., 2016; Range et al. 2009a).

#### Social disappointment

Recently, Engelmann, Clift, Herrmann, and Tomasello (2017) proposed the novel “social disappointment” hypothesis as an alternative to inequity aversion in chimpanzees. According to this hypothesis, rejection of a lower value reward, in the presence of a better-rewarded social partner, reflects subjects’ disappointment with the human experimenter for not rewarding them as well as they could have, rather than any kind of comparison of payoffs with those of a partner. Engelmann et al. (2017) provided evidence to support this hypothesis: the proportion of refusals to perform an apparatus manipulation task was significantly greater if a human was present and responsible for distributing rewards, between the subject and a partner in an adjacent enclosure, than if rewards were delivered by a machine with no human experimenter present. Furthermore, a greater number of refusals occurred in the presence of the human experimenter when the conspecific partner was absent, indicating that refusal to participate in the experiment was not based on, or intensified by, the partner receiving a better quality reward for the same amount of work.

Inequity aversion in dogs cannot be explained by this social disappointment hypothesis alone. Dogs continued working longer in the NR condition in the absence of a partner than in the RI condition; if dogs’ refusals were due to disappointment with the experimenter for not rewarding them as well as they could have, refusals to continue working should be similar in the RI and NR conditions. This is also in line with the results of Horowitz (2012; discussed above), indicating that dogs do not express social disappointment with the human.

There is, however, evidence from social interaction experiments with dogs, which we will discuss later (see ultimate function section), indicating that dogs may have negative feelings towards the experimenter following the experience of inequity. Thus, social disappointment may occur in conjunction, but not necessarily be at odds, with inequity aversion. Furthermore, some differences between the experimenter present and absent versions of the buzzer task with pet dogs indicate that subjects may respond differently in these tasks depending on whether a human is present (Brucks, Marshall-Pescini, et al., 2017).

#### Summary

Inequity aversion in animals has generated significant debate with many alternative hypotheses for primates’ negative responses in inequity conditions being proposed. All of the alternative hypotheses suggested to date as potential explanations for the negative responses in inequity tasks have been addressed and ruled out in the inequity studies with dogs.

### Cognitive prerequisites for, and mechanisms underlying, inequity aversion

A respectable proportion of papers on inequity aversion address alternative explanations for responses in inequity paradigms (as discussed in the previous section), but very little attention has been given to investigating the psychological mechanisms, prerequisites, and processes involved in inequity aversion itself. The lack of attention devoted to this topic is perhaps understandable given the doubt that inequity aversion, as opposed to the alternative explanations outlined above, can explain negative responses of animals in inequity conditions. Nevertheless, the evidence from at least some studies with primates and dogs indicates negative responses specifically to inequity, and some cognitive prerequisites have been suggested.

#### Prerequisites

In order to exhibit inequity aversion, individuals must be able to (i) recognise inequity and (ii) respond to it. The ability to perceive a relation between relations was highlighted as a necessary cognitive prerequisite for inequity aversion (Dubreuil et al., 2006); an individual needs to be able to compare the relation between their own effort and reward with that of the partner. Additionally, discriminative abilities such as numerical, quantity, or quality discrimination are surely required, to determine that the reward received by the partner, or the effort they invested, differs from one’s own. Furthermore, other non-social reward mechanisms such as reference-dependence and loss aversion have also been suggested as cognitive processes involved in inequity aversion (Chen & Santos, 2006).

Recognising inequity through these cognitive capacities must then trigger negative emotions, which drive a behavioural response. Emotions such as anger, disgust, sadness or even surprise have been proposed to be important for responses to inequity in humans. In support of the importance of emotions, brain imaging studies with humans have revealed that greater activity in the right anterior insula, part of the brain involved in processing negative emotions, is associated with increased rejection of unfair offers in economic experiments (Cheng et al., 2015; Sanfey, Rilling, Aronson, Nystrom, & Cohen, 2003; Takagishi et al., 2009). Furthermore, rejection rates increase when sadness or disgust are induced (Harlé, Chang, van ‘t Wout, & Sanfey, 2012; Harlé & Sanfey, 2007; Moretti & di Pellegrino, 2010).

Interestingly, a recent functional magnetic resonance imaging (fMRI) study revealed that aggressive dogs experience increased amygdala activation, indicative of covert arousal, while witnessing their owner providing food to a fake dog compared with witnessing their owner placing the food in a bucket (Cook, Prichard, Spivak, & Berns, 2017). Although a stronger link to inequity aversion is required before conclusions about the neural or psychological mechanisms underlying inequity aversion in dogs are drawn, this study does at least highlight methods available for addressing such issues in dogs.

Inhibitory control, or the ability to delay gratification, may also be required for an animal to sacrifice rewards to properly express their aversion. Only one study so far has demonstrated a relationship between inequity aversion and inhibitory control in a non-human animal species. This was, in fact, a recent investigation with dogs (Brucks, Range, & Marshall-Pescini, 2017). Brucks, Range, and Marshall-Pescini (2017) conducted a battery of five inhibition tests, and an impulsivity questionnaire filled out by the dog owners, and related their results to the performance of those subjects in the paw task. Dogs that were more compulsive (those that stuck with their initial choices independent of feedback) in the inhibition tests, gave their paw more in the paw task, independent of the condition. These dogs were, therefore, less likely to respond negatively to inequity. Dogs that had a slower decision speed also refused to give their paw earlier in the two inequity conditions (RI and QI) and the FC condition designed to control for individual food expectation. Dogs with lower persistence in the inhibition tests exhibited a stronger reaction specifically to the reward inequity condition, giving the paw fewer times. Furthermore, dogs with greater impulsivity, according to the owner questionnaire, also complied fewer times in the RI condition. Overall, these results provide a novel insight into the aspects of inhibition that might be important for inequity aversion, while they also lend support to the notion that inhibitory control may constrain expression of inequity aversion and may explain individual differences (Brucks, Range, & Marshall-Pescini, 2017).

Finally, if inequity aversion applies to direct reciprocity (Brosnan & Bshary, 2016; Stevens & Hauser, 2004), memory may also be involved, as an individual must compare what they gave with what they received, with a time delay in between these events.

Overall, investigations into the prerequisites and mechanisms involved in inequity aversion, especially in non-human animals, are not plentiful. The possible cognitive and emotional processes outlined here remain largely speculative as a consequence.

#### Possible simple mechanisms underlying inequity aversion

Interestingly, the cognitive prerequisites suggested for inequity aversion overlap almost entirely with the cognitive prerequisites suggested to be necessary for the evolution of reciprocity in non-human animals (Stevens & Hauser, 2004), implying an intricate link between the two processes. This cognitively complex view of reciprocity is, however, facing increasing doubt in light of growing support for simple hypothesised proximate mechanisms that have limited need for such cognitive sophistication (Brosnan & de Waal, 2002; Evers, de Vries, Spruijt, & Sterck, 2015; Evers, de Vries, Spruijt, & Sterck, 2016; Schino & Aureli, 2017). Perhaps inequity aversion, too, is driven by simpler mechanisms.

The mechanisms responsible for dogs’ inequity aversion in particular might be relatively simple given that they exhibit an apparently more primitive form of inequity aversion than other animals. In fact, Horowitz (2012) proposed that simple extinction facilitated by the reward being given to the partner, rather than inequity aversion, could explain cessation of paw-giving in the original paw task (Range et al. 2009a). However, this interpretation of extinction is still consistent with the initial conclusion that dogs display a primitive form of inequity aversion. Declining to cooperate, or to perform a behaviour, need not involve sophisticated cognitive abilities in order for it to be adaptive.

Socially mediated food expectation, as previously recognised (e.g. Brosnan et al., 2011; Brosnan et al., 2010; Hopper et al., 2014), and as mentioned earlier, might underlie inequity aversion. A subject might expect that they should, or will, receive the better reward *because* the partner is receiving it. Their subsequent refusals to perform the task may result from ensuing violation of expectation. Socially mediated food expectation could develop through the complex cognitive processes listed above such as the ability to perceive a relation between relations (Dubreuil et al., 2006) and, particularly, social reference-setting proposed by Chen and Santos (2006). However, socially mediated food expectation could also represent a simple mechanism, obviating the need for some, more complex, cognitive capacities; a subject could develop expectations of what they will receive in a social setting through associative learning.

In addition to socially mediated food expectation, more basic physiological processes might also play a role in governing responses to inequity. The presence of a feeding conspecific may stimulate normal physiological responses to feeding such as cephalic phase responses (CPRs). CPRs are physiological responses to sensory signals such as the sight and smell of food, and they elicit secretion of acid in the stomach (Feher, 2017; Ferriday & Brunstrom, 2011; Smeets, Erkner, & de Graaf, 2010). Importantly, these physiological responses can be conditioned and, therefore, could be stronger in a social setting (Power & Schulkin, 2008). These responses may ultimately induce stress in the absence of food ingestion and cause dogs to discontinue in the inequity task as a stress-avoidance mechanism.

It is worth raising the possibility that such a primitive mechanism might also play a role in more sophisticated forms of inequity aversion. For example, CPRs in a subject might also be stronger in the presence of a partner consuming a better quality reward. This could limit the extent to which a lower value reward satisfies the subject. In this regard, understanding the processes underlying primitive forms of inequity aversion in dogs might provide useful insights into the processes that underlie complex forms of inequity aversion observed in other species.

It is important to note that, even if simple mechanisms do, in fact, account for inequity aversion in dogs, and other non-human animal species, the functional aspect of such a potentially convergent trait might still be similar.

### Factors that influence inequity aversion

Numerous factors have been identified in inequity studies with non-human animal species that may influence the expression of inequity aversion either due to effects specifically in inequity conditions or due to effects across conditions more generally. These include sex (chimpanzees; Brosnan et al., 2010; Hopper et al., 2014; squirrel monkeys Talbot et al., 2011; Freeman et al., 2013; marmosets; Mustoe et al., 2016), dominance rank (chimpanzees; Bräuer et al., 2006; Brosnan et al., 2010; long-tailed macaques; Massen et al., 2012), relationship quality or length (chimpanzees; Brosnan et al., 2005; see also Hopper et al., 2014; Brosnan et al., 2015; marmosets; Mustoe et al., 2016), personality (chimpanzees; Brosnan et al., 2015), age (rhesus macaques; Hopper et al., 2013), and effort (chimpanzees; Brosnan et al., 2010; long-tailed macaques; Massen et al., 2012; capuchin monkeys; van Wolkenten et al., 2007; carrion crows and ravens; Wascher & Bugnyar, 2013).

In dogs, a number of factors that might influence inequity aversion have also been identified. Range, Leitner, and Virányi (2012) investigated whether motivation, relationship quality, and attention correlate with performance in the paw task. Interestingly, general motivation, as measured by persistence in a problem-solving task, correlated positively with performance in the NR control condition but not the reward inequity condition, which could indicate that different factors drive the dogs’ responses in these two conditions. No relationship was found between performance in the reward inequity condition and the extent to which subjects pay attention to their partner in a local enhancement task.

Relationship quality did not relate to responses in the paw task; however, dogs in more affiliative relationships (based on whether they slept in regular body contact) actually required more paw *commands* in the RI condition (Range et al., 2012). This suggests that they had a greater dislike for the inequity. The direction of this result is quite surprising as it contrasts with results from chimpanzees; with chimpanzees, the stronger the relationship between the partners, the less sensitive they were to inequity (Brosnan et al., 2005).

This result in dogs is, however, in line with the recent finding that male marmosets only display an aversion to inequity within their pair-bonds (Mustoe et al., 2016). Additionally, humans were shown to have a greater dislike for unfair offers from friends than from strangers in an economic experiment (Wu, Leliveld, & Zhou, 2011). It is, of course, possible that stronger relationships are, in fact, based on intolerance for inequity. Nevertheless, this result in dogs only relates to the paw commands and not the final paw count. Furthermore, Range et al. (2012) also investigated relationship quality in the context of co-feeding (or “food tolerance”) and found no correlation with performance in the paw task. Thus, future studies are required to clarify any effects of relationship quality on inequity aversion in dogs and to investigate what aspects of the relationship are measured with these different methods.

Interestingly, Range et al. (2012) did not find any effect of dominance rank on inequity aversion in pet dogs in the paw task. However, Essler et al. (2017), in contrast, recently reported an effect of rank in pack-living dogs tested in the buzzer task. Dominant animals had a stronger reaction to inequity in the RI condition. This effect was more intense the greater the rank distance between the subject and subordinate partner.

Different explanations might account for the conflicting results relating to effects of dominance between Range et al. (2012) and Essler et al. (2017). Assessment of the dominance rank of pet dogs in Range et al. (2012) was based on the owner’s report in a questionnaire whereas rank in the pack-living dogs was based on formal continuous observation; this difference in data collection method might have influenced the discrepancy in the result. Alternatively, the pack-living dogs may have formed stronger hierarchies, than the pet dogs, due to the presumably more limited influence of humans, who usually take over the leading role with their pets and interfere in conflicts between them. Consequently, there may have been a sufficient difference in rank, only in the pack-living dogs, for rank effects to be observed; in the absence of strong rank differences, any potential effects of rank may be negligible.

#### Factors influencing *quality* inequity aversion in dogs

Dogs have not yet shown strong negative reactions to inequity relating to the quality of rewards distributed in inequity experiments. In the paw task studies, subjects continued giving their paw to the human experimenter as long as they received some type of food, even if the partner received the higher value reward. It is currently unclear why this was the case. Here, we discuss some possible explanations for the absence of quality inequity aversion in dogs.

One must consider the possibility that dogs do not really discriminate food types in the first place, and that this explains their lack of response. However, this seems quite implausible; multiple studies demonstrate food preferences, or discrimination of food types, in dogs (Araujo & Milgram, 2004; Bhadra & Bhadra, 2014; Brucks, Soliani, Range, & Marshall-Pescini, 2017; Houpt, Hintz, & Shepherd, 1978). Furthermore, Brucks et al. (2016) selected two food types of different quality for each dyad, individually, based on the owners’ subjective assessments, which were then validated for each dog with a food preference test, prior to initiation of the inequity experiment. Nevertheless, while the dogs were capable of discriminating the two food types and preferred one over the other, we do not know the *extent* to which these rewards differed in value for the dogs. The foods may not have differed sufficiently in value to result in discontentment with receiving the non-preferred food type over the preferred. Thus, the distribution of food rewards in the QI condition may have been perceived as more or less equitable by the dogs.

Another potential explanation for the lack of an aversion to inequity in relation to reward quality, is that dogs were simply inattentive or insufficiently attentive to their partner’s rewards and interactions with the experimenter. This would be surprising given the ample evidence from cooperative problem-solving (Bräuer, Bös, Call, & Tomasello, 2013; Naderi, Miklósi, Dóka, & Csányi, 2001; Ostojić & Clayton, 2014) and social learning (Fugazza, Pogány, & Miklósi, 2016; Mersmann, Tomasello, Call, Kaminski, & Taborsky, 2011; Miller, Rayburn-Reeves, & Zentall, 2009; Range, Huber, & Heyes, 2011; Range, Virányi, & Huber, 2007; Range & Virányi, 2013; Topál, Byrne, Miklósi, & Csányi, 2006) studies indicating that dogs are capable of successfully paying attention to, and extracting information from, human and conspecific partners. Furthermore, there are even indications that dogs extract information from social interactions between third parties (Anderson et al., 2017; Carballo et al., 2015; Carballo, Freidin, Casanave, & Bentosela, 2017; Freidin, Putrino, D’Orazio, & Bentosela, 2013; Kundey et al., 2011; Marshall-Pescini, Passalacqua, Ferrario, Valsecchi, & Prato-Previde, 2011; Rooney & Bradshaw, 2006). Moreover, as mentioned above, Range et al. (2012) failed to find a link between attention to the partner, in a local enhancement task, and performance in the paw task.

It may be the case, however, that, in the paw task setting, subjects were more focused on the experimenter and/or the bowl of food such that they were not too attentive to their partner. In fact, their attentiveness to the events relating to their partner may have only peaked in the RI condition *because* they themselves were not being rewarded, and this increase in attention may, in turn, have facilitated their perception of the inequity. In support of this, Brucks et al. (2016) reported significantly longer gazing at their conspecific partner, per trial, in the RI condition compared with the ET condition, while gazing duration in other social conditions did not differ from the ET condition. This is, however, weak evidence for the current hypothesis; increased gazing at the partner in the RI condition could also reflect a violation of expectancy following perception of inequity. Furthermore, Range et al. (2009a) observed no differences in gazing across the social conditions. Nevertheless, gazing is perhaps a crude measure of attentiveness; thus, whether attentional factors can explain a lack of inequity aversion relating to quality, requires further exploration.

A final possibility is that, regardless of their ability to discriminate food types, and their attentiveness to the interactions between the human and the partner, they were not able to distinguish between the food types distributed in the experimental setting. Rewards were specifically held up between both dogs before being distributed, to aid perception; however, we cannot be certain that this act functioned as intended. Discriminating between receipt of a reward versus receipt of no reward is undoubtedly an easier feat, potentially explaining subjects’ detection of inequity, and their associated refusal to continue working, in the RI condition.

These proposed explanations for the lack of quality inequity aversion in dogs suggest that dogs simply did not perceive the inequity or that they did not view the distribution as inequitable. However, these suggestions are actually in conflict with recent evidence indicating that, even though the dogs do not react negatively to quality inequity during the paw task, they may actually perceive the situation as inequitable (Brucks et al., 2016; discussed below under ultimate function). Furthermore, Essler et al. (2017) did provide some evidence for this more advanced form of inequity aversion in dogs in the buzzer task. Thus, alternative hypotheses are needed to account for the standard absence of a negative response to quality inequity in dogs (Brucks et al., 2016; Brucks, Marshall-Pescini, et al., 2017; Range et al. 2009a).

The design of the inequity experiments carried out with dogs, to date, might limit the potential to observe the more sophisticated form of inequity aversion due to the creation of a ceiling effect. The maximum number of times subjects could give their paw, or press the buzzer, in all conditions, was capped at 30. In general, dogs reached this number in the ET and QI conditions, creating the impression that they were not averse to this inequity. However, it is entirely conceivable that, in the absence of such a restriction on the maximum count, a difference would emerge between these two conditions, allowing us to observe an aversion to inequity with respect to reward quality.

The receipt of any food reward at all may be more important to dogs than the relative value of those food rewards. Refusing to continue with the task in the QI condition in which they receive food may, therefore, have been more difficult for the dogs than refusing to continue in the RI condition in which they receive no food reward. Moreover, dogs may suffer a compulsion to comply with the human’s commands, a compulsion more difficult to resist while receiving food.

Future investigations are required to determine the reason for a general lack of response to quality inequity in these paradigms with dogs. Importantly, the factors mentioned here that may have contributed to the absence of quality inequity aversion in dogs are worth taking into consideration for other non-human animal species that fail to demonstrate sensitivity to inequity in similar paradigms.

#### The influence of humans on inequity aversion in dogs

Although the ability to respond negatively to inequity is clearly not a result of domestication, the expression of inequity aversion in dogs may be facilitated, and highly modified, by experience with humans (i.e. human exposure hypothesis: Hare, Brown, Williamson, & Tomasello, 2002) for a number of reasons. First, it has been suggested that dogs have evolved a unique set of socio-cognitive skills for living with humans, including the ability to use human communicative cues (Hare et al., 2002; MacLean, Herrmann, Suchindran, & Hare, 2017; Miklósi et al., 2003; Miklósi & Topál, 2013; Topál et al., 2009; but see Heberlein, Turner, Range, & Virányi, 2016; Lampe, Bräuer, Kaminski, & Virányi, 2017; Range & Virányi, 2013). They have even been found to attend more to humans than to dogs or non-social stimuli (Range, Horn, Bugnyar, et al., 2009b; Wallis et al., 2014) and are also able to learn socially from humans (Fugazza, Pogány, & Miklósi, 2016; Huber et al., 2009; Kubinyi, Topál, Miklósi, & Csányi, 2003; Pongrácz, Miklósi, Timár-Geng, & Csányi, 2004; Range & Virányi, 2013). Although some of these skills may have been inherited from wolves (Range & Virányi, 2015; Range & Virányi, 2013) and may, in fact, be shared with other domesticated species (Maros, Gácsi, & Miklósi, 2008; Nawroth, Baciadonna, & McElligott, 2016; Schuetz, Farmer, & Krueger, 2017), such abilities combined with lifelong, daily exposure to humans may contribute to the development of responses to inequity or even the ability to recognise inequity.

Second, as mentioned above, humans may influence the dogs’ dominance relationships through interference. Such interference might, for example, enhance the propensity of subordinate dogs to respond to inequity, while suppressing the stronger reactions of dominant individuals.

Third, overlapping with effects on hierarchical relationships, interactions with humans might impact the strength or quality of affiliative relationships between dogs. This might, in turn, influence the degree to which subjects are willing to tolerate inequity. Moreover, it may affect the degree to which they expect to be treated equitably by a human.

Finally, many pet dogs experience extensive training by their owners. In fact, a basic prerequisite for participation in the paw task is that dogs have already been trained to give their paw on command. Training by humans is likely to have a major impact on the expression of inequity aversion in dogs. In particular, we would expect that dogs trained to work without rewards, or to be more persistent, would be less likely to refuse to comply with humans. Consequently, some dogs may actually register the inequity and experience negative emotions but, due to their training, do not react. Although, this is arguably one of the strengths of the paw task: dogs presumably must act against trained impulses in order to express their negative feelings.

Of course, many of the impacts of humans, outlined here, might also apply to other captive animals. However, for dogs, these effects may occur in “real life” and may, therefore, have consequences for the function of the behaviour (see discussion of ultimate function, below).

### Summary

In summation, hypotheses including the mere presence of the better quality reward, food expectation, successive negative contrast, and social disappointment have been proposed as alternative explanations for the negative responses in inequity conditions of inequity studies. These have been controlled for and ruled out in inequity studies with dogs. The psychology involved in inequity aversion is a greatly neglected area. Certain cognitive processes have been suggested to play a role in inequity aversion. The only study so far to examine any of these was a study on the relationship between inhibitory control and inequity aversion in dogs, which indicated that certain aspects of inhibitory control do influence responses to inequity. Simple mechanisms might account for responses to inequity in dogs and other non-human animals. Furthermore, rank seems to influence the expression of inequity aversion in dogs, while relationship quality may also have an effect. Moreover, several aspects of dogs’ experience living closely with humans may influence their reactions to inequity. Finally, detection of quality inequity aversion in dogs may have been constrained by the restricted count in the experiment design, combined with satisfaction with receiving any reward and a compulsion to comply with the human.

## Ultimate function

### What is the function of inequity aversion?

The prevailing hypothesis regarding the ultimate function of inequity aversion posits it as a mechanism that stabilises cooperation among unrelated individuals (Brosnan, 2011; Fehr & Schmidt, 1999, 2006). This effect on cooperation may be achieved through punishment of non-cooperators by inequity averse individuals; evidence from economic experiments with humans indicates that active, costly punishment, or the threat thereof, suffices to achieve full cooperation (Fehr & Gächter, 2000, 2002; Fowler, 2005; Henrich et al., 2006; Saaksvuori, Mappes, & Puurtinen, 2011), while inequity aversion may function as the underlying motivation driving such punishment (Raihani & McAuliffe, 2012).

Views on how inequity aversion influences cooperation, at least in non-human animal species, are changing though. Rather than subjects’ inequity aversion motivating punishment of non-cooperators, inequity aversion may simply facilitate avoidance of such partners or withdrawal from such relationships. This view is supported by the scarcity of evidence for punishment, as a response to cheating, among animal species (see Riehl & Frederickson, 2016 for review). The avoidance of the human experimenter and the avoidance of, and reduced tolerance for, conspecific partners, observed in dogs, following inequity, also supports this emerging view (see Brucks et al., 2016 and Essler et al., 2017; discussed below). Additionally, Melis, Hare, and Tomasello (2009) demonstrated chimpanzees working to develop equitable relationships in a cooperation experiment. Inequity aversion may allow individuals the opportunity to find alternative partners and, therefore, the potential to develop more equitable cooperative relationships (Brosnan, 2013; Brosnan & de Waal, 2014; Brosnan, 2011; Brosnan & Bshary, 2016).

### Evidence that inequity or inequity aversion influences cooperation in non-human animal species

Although there is some phylogenetic support for the hypothesised role of inequity aversion in maintaining cooperation (Brosnan, 2011), surprisingly few studies have demonstrated (or investigated) an effect of inequity or inequity aversion on cooperative interactions in supposedly inequity averse species. Token-exchange and related tasks that are typically used to demonstrate inequity aversion, tend not to constitute cooperative interactions between the two rewarded individuals but rather superficial cooperation between the participant and the experimenter.

Some studies have provided evidence for a direct relationship between inequity aversion and cooperation. For example, using a counterweighted tray that pairs of capuchin monkeys had to pull together to obtain food rewards, Brosnan, Freeman, and de Waal (2006) demonstrated that the pulling success rate across conditions was significantly lower if one individual in the pair consistently dominated the higher value rewards. Additionally, de Waal and Davis (2003) demonstrated that capuchin monkeys were less likely to cooperate if rewards were monopolisable, while de Waal and Berger (2000) found that capuchins were significantly less likely to cooperate if the tray was only baited on one side, with some indication that the disadvantaged individual might have cooperated more if they received some of the rewards.

Experiments with chimpanzees also indicate a potential role for inequity aversion influencing cooperative outcomes. Pairs of chimpanzees successfully negotiated an outcome in a cooperative negotiation game in which they could choose between cooperating for an equal or heavily imbalanced reward distribution (Melis et al., 2009), while they were also shown to employ various enforcement mechanisms to mitigate freeloading in cooperative experiments (Suchak et al., 2016).

Further evidence for inequity or inequity aversion influencing cooperative outcomes comes from research on birds. In a loose-string cooperative problem-solving task, Massen, Ritter, and Bugnyar (2015) demonstrated that ravens’ likelihood of cooperating was lower after experiencing displacement by their partner that resulted in inequity. Similarly, in a cooperative string-pulling task with kea, a trend emerged whereby dyads were more likely to attempt cooperation following equitable division of rewards in the previous trial (Schwing, Jocteur, Wein, Noë, & Massen, 2016). Interestingly, despite this evidence that equity might influence kea’s decisions to cooperate, recent findings using a token-exchange task suggest that kea are not inequity averse (Heaney et al., 2017), questioning the comparability of cooperation tasks and the standard inequity tasks.

Recent research on dogs adds to the evidence supporting a relationship between inequity aversion and cooperation. Immediately after each condition of the paw task, Brucks et al. (2016) conducted a food tolerance test in which the pair of dogs were permitted to simultaneously eat from a food bowl containing slices of sausage. Dogs spent less time co-feeding in tolerance tests that directly followed the RI (inequity) condition compared with those that followed the ET (equity) condition. Importantly, monopolisation of the food, by the subjects, disadvantaged by the inequity immediately prior to the test, seemed to drive this outcome, as their duration of “feeding alone” increased relative to that in tests following the ET condition. Moreover, Brucks et al. (2016) observed parallel effects of *quality* inequity on subsequent tolerance, with subjects spending significantly less time co-feeding after the QI condition than after the ET condition. This particularly striking result strengthens the claim that inequity, rather than response to lack of individual rewards, negatively impacts dogs’ subsequent tolerance for partners, as subjects themselves received, on average, the same quality and amount of rewards in the ET condition as they did in the QI condition.

Perhaps even more intriguingly, this result suggests that subjects noticed the quality inequity and this did, in some way, affect them, with consequences for their subsequent social interactions. In fact, if it is truly inequity rather than other reward-schedule effects driving this response, it potentially represents an interesting strategy: dogs accrue the available benefits during the paw task, avoiding costs associated with giving up, and only later act on the inequity they suffered, once they have the power to influence the outcome.

Tolerance for partners is particularly important in the context of cooperation. It is considered a prerequisite for cooperation and has been shown to correlate positively with cooperative success for numerous species tested in cooperative problem-solving tasks including chimpanzees (Melis, Hare, & Tomasello, 2006), Barbary macaques (*Macaca Sylvanus*; Molesti & Majolo, 2016), marmosets (Werdenich & Huber, 2002), ravens (Massen et al., 2015), and rooks (*Corvus frugilegus*; Seed, Clayton, & Emery, 2008). Given this apparent relationship between tolerance and cooperation, the reduced tolerance observed by Brucks et al. (2016), following inequity, suggests that inequity has the potential to negatively influence cooperation in dogs. Of course, tolerance or food-sharing in this manner could be viewed as a cooperative investment (Noë, 2006) and may, therefore, be the critical cooperative context in which inequity and an aversion thereto plays a role.

Immediately after the food tolerance tests, Brucks et al. (2016) monitored proximity-seeking in the subjects. The experimenter and owner knelt on the floor of the test room, approximately 2 meters apart, for 10 minutes while the two dogs were free to roam around and interact with each other, the experimenter, or the owner. Subjects took significantly longer to approach the experimenter, and spent significantly less time in close proximity to their conspecific partner, following the RI condition compared with the ET condition. Importantly, this test was also carried out after the NR condition and no differences between the ET and NR conditions emerged; therefore, inequity, rather than the simple absence of reward, is likely to explain the results observed following the RI condition.

Similarly, Essler et al. (2017) found that after the RI condition, subjects spent significantly less time in proximity to their conspecific partner, compared with after the ET condition. Unlike in Brucks et al. (2016), the RI condition seemed to have no effect on proximity-seeking directed towards the experimenter. Interestingly, however, latency to approach the experimenter after the QI condition exceeded that of the ET condition. Furthermore, the duration of time spent in proximity to the experimenter after the QI condition was significantly lower than that after the ET condition. Apart from supporting potential effects of inequity on cooperation in dogs, these results also reinforce the idea that dogs recognise inequity related to differences in reward quality. Furthermore, it suggests that there may be some negative emotions towards the experimenter which is partially in line with the social disappointment hypothesis (Engelmann et al., 2017).

### What is the function of inequity aversion in dogs?

Dogs are considered by many to be a cooperative species (Bonanni & Cafazzo, 2014; Hare & Tomasello, 2005; Hare et al., 2002; Miklósi & Topál, 2013; Naderi et al., 2001). Evidence from numerous sources supports this view. For example, recent laboratory experiments with dogs demonstrated that they provision familiar conspecifics with food in a tray-pulling (Quervel-Chaumette et al., 2015) and a token-choice paradigm (Dale, Quervel-Chaumette, Huber, Range, & Marshall-Pescini, 2016). Additionally, dogs have recently been shown to cooperate by generalised reciprocity and possibly also direct reciprocity (Gfrerer & Taborsky, 2018; Gfrerer & Taborsky, 2017). Moreover, dogs succeeded in coordinating with conspecifics and humans in the classic loose-string paradigm (Ostojić & Clayton, 2014; but see Marshall-Pescini, Schwarz, Kostelnik, Virányi, & Range, 2017), as well as with conspecifics in a novel cooperative problem-solving task requiring coordination (Bräuer et al., 2013). Additionally, dogs display diverse capabilities and unique cooperative potential when working with humans as herding dogs, hunting dogs, guide dogs, and more (Gácsi, McGreevy, Kara, & Miklósi, 2009; Naderi, Miklósi, & Dóka, 2002; Wobber, Hare, Koler-Matznick, Wrangham, & Tomasello, 2009).

Inequity aversion could, surely, influence cooperation in many of the situations listed above, particularly those involving food. However, it is worth questioning how realistic some of these laboratory-based examples of cooperation are, or how relevant they are to everyday social interactions among dogs.

#### Inequity aversion in cooperative interactions with humans

Daily interactions with humans might provide a setting in which inequity aversion plays a role for dogs, especially given that these interactions involve provisioning of food. The opportunity to find new cooperative human partners is unlikely to be an option for dogs; thus, refusal to comply with the human’s commands could be considered a form of punishment for the human, particularly in a working context, thereby promoting future cooperative behaviour.

It is interesting to note that the standard triadic inequity paradigms, in which a human experimenter distributes food to two non-human animals, is more ecologically and socially valid for pet dogs than it is for any other non-human animal species, as only dogs interact regularly with humans in this manner.

If inequity aversion is important for cooperative interactions with humans, we might expect breed differences in responses to inequity given that differences exist in the extent to which various breeds were selected for close cooperation with humans (Gácsi et al., 2009). In fact, in relation to our discussion of potential human influences on inequity aversion in dogs, breed differences are all the more likely given that breeds also differ in trainability (Serpell & Hsu, 2005; Turcsán, Kubinyi, & Miklósi, 2011) and the ability to use human communicative cues (Gácsi et al., 2009).

The idea of inequity aversion in dogs being of functional significance in cooperative interactions with humans is, at the same time, quite counterintuitive and perhaps implausible. Working dogs that are highly sensitive to inequity are surely undesirable from the human’s perspective. It would seem reasonable to speculate that inequity aversion was selected against; yet, dogs refuse to comply with a human’s command under conditions of inequity. In this context, though, it is important to re-emphasise that dogs only display a rudimentary form of inequity aversion. Advanced forms of inequity aversion, as seen in primates, may have been selected against in dogs *because* they were undesired by humans in working contexts. Thus, inequity aversion might be expected to have limited impact in cooperative interactions with humans due to the effects of artificial selection. In this regard, comparing inequity aversion in dog breeds with different selection histories seems imperative.

#### Inequity aversion in cooperative interactions among free-ranging dogs

Cooperation also occurs among free-ranging dogs and may provide a setting in which inequity aversion is important. In fact, it was recently argued that dogs’ social ecology, in addition to selection by humans, should be taken into consideration to more fully understand dog behaviour (Marshall-Pescini, Cafazzo, Virányi, & Range, 2017).

Cooperative hunting has been observed in free-ranging dogs; however, it is not a commonly reported behaviour, with free-ranging dogs relying primarily on scavenging of human refuse (Krauze-Gryz & Gryz, 2014; Lord, Feinstein, Smith, & Coppinger, 2013; Majumder et al., 2014; Ruiz-Izaguirre et al., 2015; Silva-Rodríguez & Sieving, 2011, 2012; Vanak & Gompper, 2009). Furthermore, evidence from recent studies of captive pack-living dogs suggests that food-sharing as well as coordinated cooperation in a food context is highly constrained in dogs (Dale, Range, Stott, Kotrschal, & Marshall-Pescini, 2017; Marshall-Pescini, Schwarz, et al., 2017; Range, Ritter, & Virányi, 2015). Therefore, inequity aversion is unlikely to have a major role to play in cooperative hunting contexts for dogs.

However, cooperation among free-ranging dogs occurs in the context of group defense of puppies, territories, and food resources (see Bonanni & Cafazzo, 2014; Bonanni, Valsecchi, & Natoli, 2010). Given that free-ranging dogs cooperate in situations that do not directly lead to food rewards, it is possible that their aversion to inequity also applies to non-food contexts. There is currently no study investigating whether dogs respond negatively to inequity in non-food contexts. This would be worthwhile to investigate not only due to its potential relevance to cooperation in free-ranging dogs but also due to its potential relevance for interactions between dogs and humans; dogs may, for example, also value social rewards such as petting or human attention (see Bhattacharjee, Sau, Das, & Bhadra, 2017; Cook, Prichard, Spivak, & Berns, 2016; Feuerbacher & Wynne, 2014). Interestingly, aversion to an inequitable distribution of food rewards has been demonstrated in other species that do not cooperate in the domain of food (for example, see Massen et al., 2012), suggesting that inequity aversion may be a higher order or domain-general capacity that can be applied to different cooperative contexts.

A final consideration is that inequity aversion may have no relevance for cooperation in dogs at all. Inequity aversion may not even have any adaptive significance for dogs. Given that wolves, dogs’ closest living relatives, are highly cooperative (Mech & Boitani, 2003; Mech, Smith, & MacNulty, 2015) and inequity averse (Essler et al., 2017), inequity aversion in dogs may simply be a remnant of inequity aversion in wolves. Clearly future research is required to elucidate the ultimate function of inequity aversion in dogs, and the situations to which it applies, if any.

### The study of inequity aversion in humans vs. animals

It is important to mention that inequity aversion tasks in animals generally differ fundamentally from those in humans. Human studies generally employ the ultimatum game (Güth, Schmittberger, & Schwarze, 1982). In the ultimatum game, a proposer can decide how to split a sum of money between her/himself and a second player, the responder. If the responder rejects the offer, neither player receives any sum of money. Therefore, the payoff of the first player is contingent on the decision of the second; if the responder is dissatisfied with the offer, their rejection ensures the proposer receives nothing. In contrast, in the animal studies, refusal to accept rewards, or to participate further in the experiments, tends to have no consequences for the payoff of the partner. Consequently, a negative response to inequity in human experiments *decreases* inequity, while in animal studies rejection of offers *increases* inequity.

This difference could have implications for how the behaviour in these experiments is interpreted. For example, in human studies, rejecting negative offers could be considered a spiteful strategy. A spiteful strategy may be adaptive due to fitness-levelling effects and has been shown to be capable of evolving alongside a fair strategy in a simulation model (Forber & Smead, 2014). In fact, McAuliffe, Blake, and Warneken (2014) provided evidence for spite driving children’s rejections of disadvantageous inequity; the children only rejected offers if it reduced the partner’s payoff. However, such an explanation for rejections of disadvantageous inequity cannot be applied to animals.

Further factors have been identified that might contribute to the evolution of fair behaviour in the ultimatum game, including reputation (Nowak, Page, & Sigmund, 2000), empathy (Page & Nowak, 2002), learning (Rand, Tarnita, Ohtsuki, & Nowak, 2013), specific spatial population structures (Page, Nowak, & Sigmund, 2000), probabilistic decision making (Ichinose & Sayama, 2015), and random allocation (Wang, Chen, & Wang, 2015). Although these criteria may explain behaviour of humans in the ultimatum game, they may not be applicable to the negative responses of animals to inequity.

In this context, Henrich (2004) criticised Brosnan and de Waal’s (2003) interpretation of the capuchins’ behaviour as inequity aversion, highlighting that humans do not refuse offers unless it deprives the partner of their payoff too. Nevertheless, evidence for rejection of unfair offers in economic games does exist for humans now, even when rejection has no consequence for the partner’s payoff (see, for example Takagishi et al., 2009 and Yamagishi et al., 2009). Additionally, negative responses in animals could still represent precursors to the behaviour observed in humans (Brosnan & de Waal, 2004).

Henrich (2004) also argued that that the behaviour of capuchin monkeys does not match the predictions of Fehr and Schmidt’s (1999) inequity aversion model; the monkeys should not have rejected their reward as doing so increased inequity. The negative responses of animals in experimental settings may, nonetheless, reflect adaptive responses that would impact the partner’s payoff under more natural settings (van Wolkenten et al., 2007). Alternatively, negative responses of animals potentially reflect immediate attempts to achieve equity by altering the behaviour of the experimenter (van Wolkenten et al., 2007). Finally, given that human inequity experiments typically involve interactions between two conspecifics, whereas animal experiments involve interaction with a third-party heterospecific (and in an unnatural setting), such strict comparisons across species and studies is difficult.

A final point worth considering is that the dyadic interaction that occurs between the subject and human experimenter in exchange tasks mirrors, in some respects, that between the proposer and responder in the ultimatum game. One could view the human experimenter in animal studies as the “proposer” who has a pool of resources available to share with the “responder” i.e. the subject. It is perhaps surprising that dogs, in particular, do not react negatively when they are the only individual interacting with the experimenter; the presence of a better-rewarded conspecific seems to be necessary to elicit a negative response. In this regard, further exploration of the importance of the experimenter and partner, to the subject, in these setups, is warranted.

### Summary

Inequity aversion is thought to ultimately function in the maintenance of cooperation. Although some support for this exists at a phylogenetic level, with some cooperative species but not non-cooperative species displaying an aversion to inequity, few studies demonstrate the interplay between inequity and cooperation in non-human animals. Recent research with dogs adds to the few examples that exist, demonstrating that the experience of inequity reduces subjects’ subsequent tolerance for partners while they also avoid their partner and the human experimenter following inequity. Many examples of cooperation, with humans and with conspecifics, exist for dogs, providing potential scenarios in which inequity aversion might play a role. It is also possible, however, that inequity aversion has no ultimate function in dogs and that it was simply inherited from wolves. Finally, it is worth noting that human inequity aversion might be fundamentally different in humans than in non-human animals at least as it has been studied to date.

## Conclusion

The study of inequity aversion in non-human animal species has amassed a substantial body of work. To date, dogs are one of the best studied species in this field with multiple developments emerging in recent years. It is clear, however, that considerable gaps in our knowledge and understanding of inequity aversion in non-human animals still exists. Several questions and remaining issues emerged in this review which invite future research. For example, inequity aversion has been a particularly replicable finding in dogs; however, a recent discrepancy warrants further investigation. Furthermore, the psychological mechanisms underlying inequity aversion are very poorly understood. In particular, whether responses to inequity are based on simple physiological responses to being in the presence of a feeding partner or whether individuals actually perceive the inequity of the situation is not yet known. Dogs may be unique in this context since their inequity aversion is potentially primitive. In this regard, studying such mechanisms in dogs, might help shed light on the mechanisms involved in inequity aversion in other species, even those with more complex forms. Importantly, studying its mechanisms could certainly illuminate the field of cooperation particularly in relation to the questions of the proximate mechanisms underlying reciprocity.

Additionally, is inequity aversion in dogs really a primitive form or do practical issues relating to experimental design, such as a restricted count, limit our ability to observe more advanced forms such as a response to differences in quality? This is currently a pertinent question as multiple lines of evidence support dogs’ sensitivity to quality inequity. Moreover, this question is of relevance to the study of other non-human animal species, as their inequity aversion might also have been masked by similar methodological restrictions. Further investigating this issue in dogs could also reveal the factors that limit the expression of sophisticated inequity aversion, perhaps revealing important steps in the evolution of this behaviour.

Finally, additional avenues for future study are the questions of whether dogs would be inequity averse if rewards were not food-based. In fact, this feeds into questions relating to the ultimate function of inequity aversion in dogs. Does inequity aversion act as a mechanism that maintains cooperation in dogs, and, if so, what forms of cooperation? Furthermore, is it relevant to interspecific cooperation with humans? And, how is it modified by experience with humans? Overall, inequity aversion in dogs is ripe for future investigation.
